# Isolation, Identification and Molecular Mechanism Analysis of the Nematicidal Compound Spectinabilin from Newly Isolated *Streptomyces* sp. DT10

**DOI:** 10.3390/molecules28114365

**Published:** 2023-05-26

**Authors:** Yuchen Sun, Jin Xie, Lihua Tang, Arome Solomon Odiba, Yanlu Chen, Wenxia Fang, Xiaogang Wu, Bin Wang

**Affiliations:** 1 College of Agriculture, Guangxi University, Nanning 530004, China; 2 Institute of Biological Sciences and Technology, Guangxi Academy of Sciences, Nanning 530007, Chinawfang@gxas.cn (W.F.)

**Keywords:** plant parasitic nematode, natural products, nematicide, biological control, drug target

## Abstract

Plant parasitic nematodes (PPNs) are highly destructive and difficult to control, while conventional chemical nematicides are highly toxic and cause serious environmental pollution. Additionally, resistance to existing pesticides is becoming increasingly common. Biological control is the most promising method for the controlling of PPNs. Therefore, the screening of nematicidal microbial resources and the identification of natural products are of great significance and urgency for the environmentally friendly control of PPNs. In this study, the DT10 strain was isolated from wild moss samples and identified as *Streptomyces* sp. by morphological and molecular analysis. Using *Caenorhabditis elegans* as a model, the extract of DT10 was screened for nematicidal activity, which elicited 100% lethality. The active compound was isolated from the extracts of strain DT10 using silica gel column chromatography and semipreparative high-performance liquid chromatography (HPLC). The compound was identified as spectinabilin (chemical formula C_28_H_31_O_6_N) using liquid chromatography mass spectrometry (LC-MS) and nuclear magnetic resonance (NMR). Spectinabilin exhibited a good nematicidal activity on *C. elegans* L1 worms, with a half-maximal inhibitory concentration (IC_50_) of 2.948 μg/mL at 24 h. The locomotive ability of *C. elegans* L4 worms was significantly reduced when treated with 40 μg/mL spectinabilin. Further analysis of spectinabilin against known nematicidal drug target genes in *C. elegans* showed that it acts via target(s) different from those of some currently used nematicidal drugs such as avermectin and phosphine thiazole. This is the first report on the nematicidal activity of spectinabilin on *C. elegans* and the southern root-knot nematode *Meloidogyne incognita*. These findings may pave the way for further research and application of spectinabilin as a potential biological nematicide.

## 1. Introduction

Plant parasitic nematodes (PPNs) invade and parasitize the host plant in three major ways: endoparasitic, semi-endoparasitic or semi-exoparasitic and exoparasitic. PPNs obtain nutrients from plants, causing serious mechanical damage and infections to the plants [1,2]. Infections by these parasites are major causes of plant diseases and crop losses, which was estimated to be 157 billion US dollars in damage to food and fiber crops worldwide every year [3]. Phytoparasitic nematodes can survive, reproduce and spread fast under various environmental conditions. They can colonize and survive in many plant parts, including the root, stem and the leaf. These characteristics make PPNs the most difficult crop pests to control [4]. Several methods are commonly used to control PPNs, including biological control, cultural control and chemical control [5].

Due to increasing concerns about human health and environmental protection, many synthetic formulations for phytoparasitic nematode control are progressively being dismissed. Furthermore, resistance to anthelmintics is common among nematodes, and the availability of effective anthelmintics is limited [6]. Therefore, the search for alternative strategies, including the use of natural metabolites, has become very important. Avermectin is the most widely and frequently used nematode control agent, but drug resistance has to be overcome [7]. In addition, the market value of nematicidal agents is increasing every year because of the frequent occurrence of nematode diseases in plants [8]. Therefore, the discovery of new, efficient and low-toxicity nematicides from biological sources such as microorganisms or plants is the key point and the major challenge in the current research for comprehensive nematode control [9]. The use of biological agents for controlling PPNs is environmentally friendly and contributes to the improvement of the soil’s ecological environment, making it an ideal nematode control measure [10].

*Streptomyces* is the main genus of actinomycetes that produces natural products effective against nematodes, making them a vital microbial resource [11]. For example, *S. avermitilis* produces the most widely used avermectin, *S. nanchangensis* produces nanchangmycin, and *S. tendae* produces nikkomycin [12,13,14]. All of these compounds can kill PPNs and have already been widely used in agricultural production. Spectinabilin (also known as neoaureothin) is another potent nematicide; it was first discovered in *Streptomyces spectabilis* in 1976 [15,16]. This drug could be produced from chorismate, five molecules of methylmalonyl-CoA, and one malonyl-CoA by polyketide synthase (PKS) [17]. Spectinabilin is one of the only two drugs (another is iodoindoles) that cause vacuolar death; however, the action mode of spectinabilin against PPNs in detail still remains unknown. In a greenhouse experiment, the survival rate of the trees could increase from 10 to 80% after using spectinabilin to treat the nematode-infected pine trees [18,19].

The PPNs, due to their complex life cycle and the difficulty in culturing, are not suitable for large-scale anti-parasitic nematode drug screening and target gene identification [20]. However, the model organism *Caenorhabditis elegans* is an invaluable tool for anti-PPNs drug discovery due to its highly evolutionary conservation with parasitic nematodes [21]. Interestingly, all known antiparasitic nematode agents have been found to be effective in killing *C. elegans*. Therefore, the *C. elegans* system is a good alternative for nematode drug screening and identification of target genes due to its ease of maintenance and molecular manipulation [22,23,24].

In this study, we used *C. elegans* as a model to screen a library of 161 bacterial isolates from moss, and we found a DT10 strain with antinematode activity. DT10 was identified as *Streptomyces* sp. by morphological and molecular analysis. The active compound isolated from strain DT10 was characterized as spectinabilin (chemical formula C_28_H_31_O_6_N) using liquid chromatography mass spectrometry (LC-MS) and nuclear magnetic resonance (NMR). Furthermore, we evaluated the effect of spectinabilin on both *C. elegans* and the southern root-knot nematode *Meloidogyne incognita*. We also investigated the nematicidal effect of spectinabilin on *C. elegans* mutants of known molecular targets.

## 2. Results

### 2.1. Screening and Identification of Strains with Nematicidal Activity

**Figure 1:**
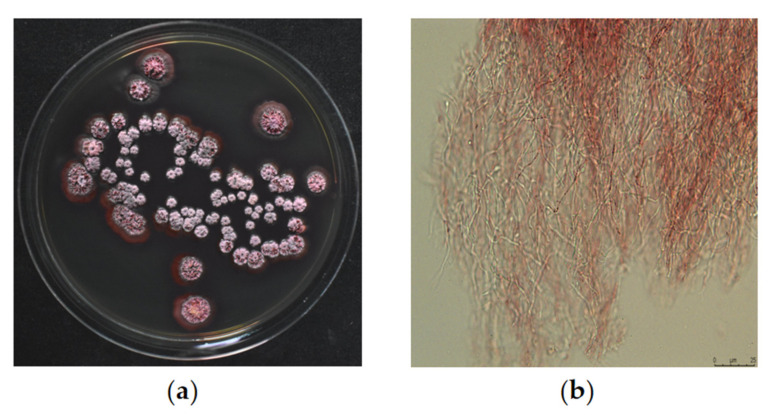
Morphological characters of DT10 strain. (**a**) Representative images of the DT10 colony on ISP2 medium. Image was photographed using a Nikon Digital Single Lens Reflex camera. (**b**) The morphology of DT10 mycelia. Image was captured with a Leica DM6B fluorescence microscope using 40× objective (scale bar = 25 µm).

A total of 161 strains were isolated from wild moss using a concentration gradient dilution method (Table S1). These strains were cultured in ISP2 plates and extracted using mixed solvent of ethyl acetate, methanol and acetic acid. The extract was then screened for nematicidal activity at the working concentration of 0.1 μg/μL for 24 h in the *C. elegans* model. The DT10 strain showed a significant nematicidal activity, reducing the viability of L1 worms by 100% compared to the control. Morphological observations at 14 days showed that DT10 grew well on ISP2 medium, with a dry and wrinkled surface of the colony and purplish red substrate mycelia (Figure 1). The aerial mycelia are difficult to pick up. The strain produced a large amount of red soluble pigment, resulting in a dark red extract obtained after soaking the medium in the organic solvents.

**Figure 2:**
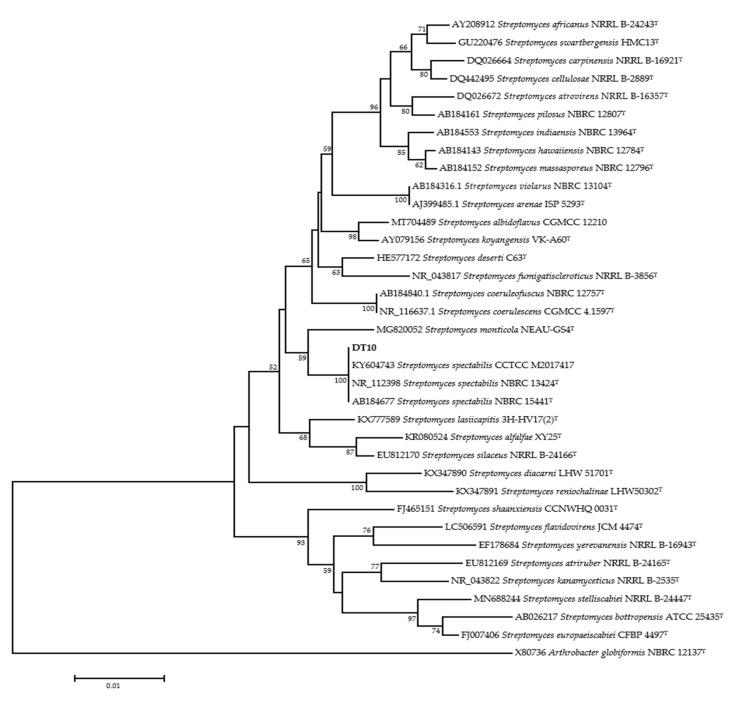
Unrooted phylogenetic tree of strain DT10 based on 16S rRNA sequences. Bootstrap values are expressed as percentages. Numbers at branching points refer to bootstrap value based on neighbor-joining analysis of 1000 resample data sets. The scale bar presents sequence divergence. The strain marked with “T” represents type strain.

Furthermore, we sequenced the 16S rRNA gene fragment of strain DT10, and a full-length sequence of 1409 bp was submitted to GenBank (accession number OQ926623). BLAST analysis showed 99.79% similarity to *S. spectabilis*. Phylogenetic analysis of the 16S rRNA sequences of 34 typical *Streptomyces* strains revealed that the strain DT10 was most closely related to *S. spectabilis* (Figure 2). Based on molecular characteristics, strain DT10 was identified as *Streptomyces* sp.

### 2.2. Nematicidal Active Compound ***1*** Extracted from DT10 Fermentation Product and Identification of Compound ***1*** as Spectinabilin

**Figure 3:**
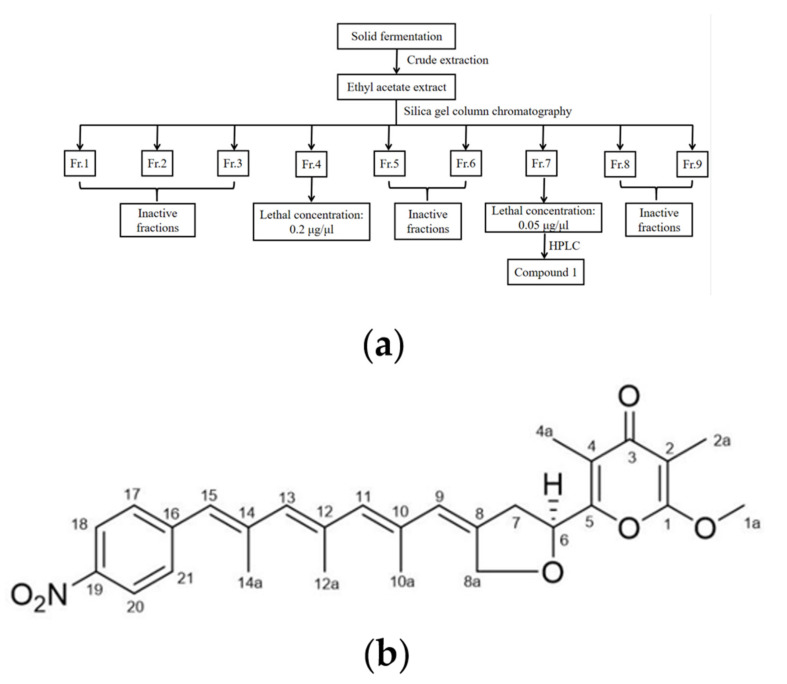
Isolation and nematicidal activity of main active compound **1** of strain DT10. (**a**) Flow chart showing extraction and separation procedure of compound **1** from strain DT10. (**b**) Chemical structure of compound **1** (Spectinabilin).

Due to the strong nematicidal activity exhibited by the crude extract of strain DT10, we next proceeded to ferment the strain DT10 and conducted crude extraction of strain DT10 to identify the active components. Guided by activity tracing, ethyl acetate extracted components were further fractionated using silica gel column chromatography and high-performance liquid chromatography (HPLC); finally, the main nematicidal active compound 1 was isolated from DT10 (Figure 3a, Table S2).

The purified compound **1** from DT10 appeared as a yellow powder and was identified using high-resolution electrospray ionization mass spectrometry (HR-ESI-MS): *m*/*z* [M + H]^+^, 478.2226 (Figure S2). Based on this information, the molecular formula of this compound **1** was predicted to be C_28_H_31_O_6_N(Cacl. for C_28_H_32_O_6_N^+^: 478.2230). ^1^H NMR (MeOD, 800MHz, Figure S3): δ 8.22(2H, d, *J* = 8.8 Hz, H-18, H-20), 7.55 (2H, d, *J* = 8.64 Hz, H-17, H-21), 6.53 (1H, s, H-15), 6.15 (1H, s, H-9), 6.01 (1H, s, H-13), 5.88 (1H, s, H-11), 5.27 (1H, dd, *J* = 5.44, 7.68 Hz, H-6), 4.84 (1H, m, H-8a), 4.75 (1H, d, *J* = 14.64 Hz, H-8b), 4.01 (3H, s, H-1a), 3.11 (1H, dd, *J* = 8.4, 17.36 Hz, H-7a), 2.96 (1H, s, dd, *J* = 5.36, 16.08 Hz, H-7b), 2.10 (3H, s, H-14a), 2.04 (3H, s, H-12a), 2.01 (3H, m, H-10a), 2.00 (3H, m, H-4a), 1.82 (3H, m, H-2a); ^13^C NMR (MeOD, 200 MHz, Figure S4): δ 181.4 (C-3), 163.2 (C-1), 156.8 (C-5), 145.9 (C-19), 144.8 (C-16), 139.3 (C-14), 137.9 (C-8), 135.5 (C-12), 134.8 (C-10, overlap), 134.8 (C-11, overlap), 134.1 (C-13), 129.5 (C-17, overlap), 129.5 (C-21, overlap), 127.8 (C-15), 126.4 (C-9), 123.1 (C-18, overlap), 123.1 (C-20, overlap), 119.1 (C-4), 99.1 (C-2), 72.9 (C-6), 69.6 (C-8a), 55.1 (C-1a), 37.5 (C-7), 18.3 (C-12a), 18.2 (C-14a), 16.6 (C-10a), 8.0 (C-4a), 5.6 (C-2a). All these data were comparable with published NMR data for spectinabilin [25]. Thus, Compound **1** was identified as spectinabilin (Figure 3b).

### 2.3. Nematicidal Effect of Spectinabilin

**Figure 4:**
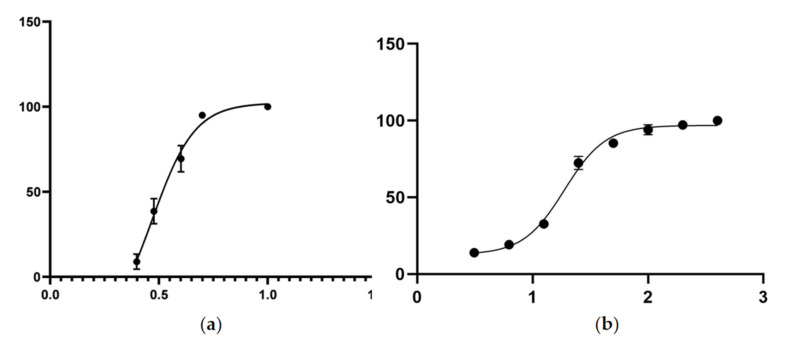
Dose–response curve of spectinabilin’s nematicidal activity on *C.elegans* L1 worms. The curve shows that the nematicidal activity was positively correlated with the concentration of spectinabilin. The assay was employed to calculate the half-maximal inhibition concentration (IC_50_) after (**a**) 24 h of incubation with spectinabilin at 2.5, 3, 4, 5 and 10 μg/mL and (**b**) 2 h of incubation with spectinabilin at 3.125, 6.25, 12.5, 25, 50, 100, 200 and 400 μg/mL. The data were analyzed using Prism 8.0.2. Data points represent experiments conducted in triplicate; mean ± standard error of the mean (SEM).

We treated *C. elegans* L1 worms with spectinabilin at working concentrations between 2.5–10 μg/mL for 24 h and analyzed the mortality of the nematodes. The results showed that the nematicidal activity of the spectinabilin was positively correlated with the concentration (Table 1). A fatality rate of 100% was observed at a working concentration of 10 μg/mL and more than 90% at 5 μg/mL, while there was a significant decline below 4 μg/mL. The nematicidal activity was almost abolished at 2.5 μg/mL. The IC_50_ value at 24 h of spectinabilin was 2.948 μg/mL according to the dose–effect curve fitting equation drawn by the logarithmic transposition of concentration (Figure 4a).

To achieve a shorter treatment duration, we increased the range of spectinabilin concentration to 3.125–400 μg/mL and evaluated its nematicidal activity on *C. elegans* L1 worms after 2 h. The results showed that the fatality rate was over 90% at 200 and 100 μg/mL and reached 100% at 400 μg/mL (Table S3). Spectinabilin appears to kill worms faster when compared with the many nematicidal compounds of microbial or plant origin that have been isolated and purified in the laboratory. Such compounds include volatile organic compounds from *Daldinia* cf. *concentrica*, chemical constituents from the fungus *Stereum* sp. YMF1.04183 and nematicidal compounds from *Pseudobambusicola thailandica* [26,27,28]. Based on the dose–effect curve fitting equation drawn by the logarithmic transposition of concentration, the IC_50_ value at 2 h of spectinabilin was calculated to be 18.86 μg/mL (Figure 4b).

### 2.4. Spectinabilin Reduces the Motility of C. elegans L4 Worms

**Figure 5:**
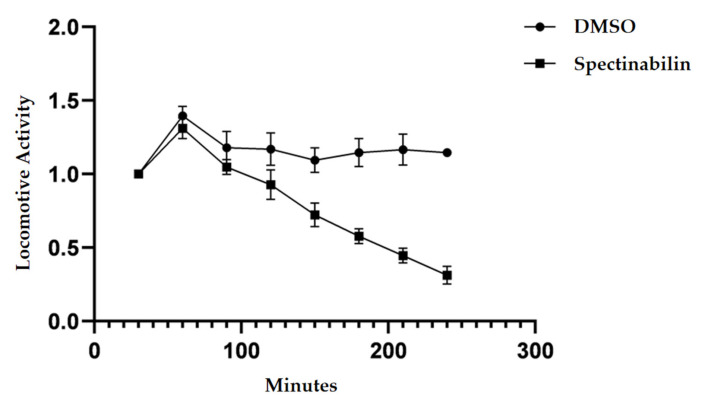
Effect of spectinabilin on locomotive ability of *C. elegans* L4 worms. Relative locomotive activity of the worms incubated with 40 μg/mL spectinabilin was captured using WMicrotracker ARENA unit and analyzed using Prism 8.0.2. Data points represent four independent experiments; mean ± standard error of the mean (SEM).

We treated *C. elegans* L4 worms with spectinabilin for 4 h at concentrations between 5 and 80 μg/mL, and the mortality was then investigated (Table S4). The results showed that the nematicidal activity at 4 h was positively correlated with the concentration above 40 μg/mL but there was no significant nematicidal activity against *C. elegans* L4 worms within the working concentration range of 5–40 μg/mL. Although spectinabilin did not kill the *C. elegans* L4 worms at the working concentration of 40 μg/mL, the rate and amplitude of *C. elegans* body oscillation were both significantly lower than those of the control group. Therefore, 40 μg/mL was used as the experimental working concentration for testing the motility of *C. elegans* against spectinabilin using WMicrotracker ARENA unit. After processing the data derived from the instrument records, the relative locomotive activity of *C. elegans* was analyzed. The results showed that spectinabilin decreased the locomotion ability of *C. elegans* L4 worms (Figure 5). Although *C. elegans* L4 worms were not directly killed by the compound, their motility gradually decreased after treatment, and the relative activity of *C. elegans* decreased to a lower level (31.25 ± 0.06%) after 4 h.

### 2.5. Analysis of Spectinabilin on Known Nematicidal Molecular Targets

**Figure 6:**
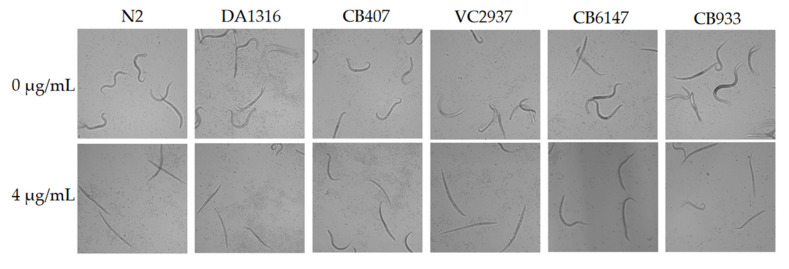
Analysis of spectinabilin on known drug target genes. The L1 worms of *C. elegans* mutants of known nematicidal drug target genes were treated with 4 μg/mL spectinabilin for 24 h. The lethality of worms was counted, and the images were captured using Leica DMi8 inverted microscope using 10× objective (scale bar = 100 μm).

Since this is the first report on the nematicidal activity of spectinabilin, we were interested in investigating whether spectinabilin functions through the known nematicidal drug targets in *C. elegans*. L1 worms of N2 and five known *C. elegans* mutants of drug target genes (DA1316, VC2937, CB407, CB6147 and CB933) were treated with 4 μg/mL spectinabilin for 24 h and observed under a microscope. The results showed that all worms survived and moved normally without spectinabilin treatment. However, N2 and all the mutant worms recorded more than 60% mortality after treatment with 4 μg/mL spectinabilin (Figure 6). These results indicated that spectinabilin had nematicidal activity on the five mutants (DA1316, VC2937, CB407, CB6147 and CB933), suggesting that the target of spectinabilin is not any of the known drug target genes investigated here and spectinabilin is possibly acting through a new class of drug targets.

### 2.6. Spectinabilin Exhibited Nematicidal Activity against Meloidogyne incognita J2s

**Figure 7:**
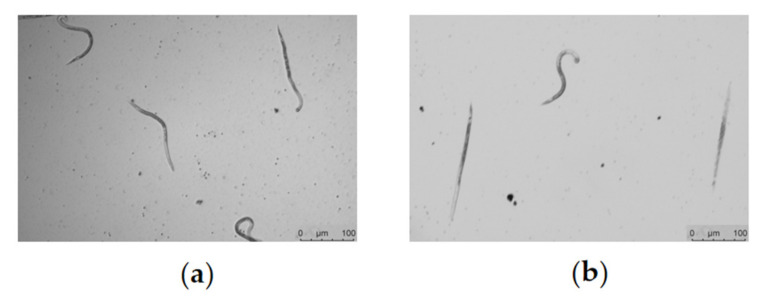
Morphological observation of *M. incognita* incubated with spectinabilin. (**a**) *M. incognita* in the control; (**b**) *M. incognita* treated with 12.5 μg/mL spectinabilin for 72 h. Images were captured using Leica DMi8 inverted microscope using 10× objective (scale bar = 100 μm).

In order to verify the nematicidal activity of spectinabilin against plant parasitic nematode *M. incognita*, J2s worms were treated with spectinabilin at working concentrations between 6.25 and 100 μg/mL for 72 h, and the mortality of the nematode was analyzed. The results showed that the nematicidal activity of spectinabilin was positively correlated with the concentration (Table 2). The fatality rate was around 40% when the working concentration was 100 μg/mL and was almost abolished at 6.25 μg/mL. The southern root-knot nematodes moved normally in the control group, while becoming straight and immobile when treated with spectinabilin (Figure 7).

## 3. Discussion

Biological control using microorganisms provides a sustainable solution for controlling the harmful effects of pests in agriculture [29]. *Streptomyces* sp. is well known for its ability to produce a large number of natural medicinal products, including antibiotics and anticancer drugs [30,31]. It accounts for the largest number and is the most widely applied genus among the microbial resources of actinomyces, which is of great value in the comprehensive prevention and control of agricultural pests [32]. The most widely used avermectin produced by *S. avermitilis*, together with nanchangmycin and nikkomycin from *S. nanchangensis* and *S. tendae*, respectively, can kill nematodes [12,13,14]. All the above compounds are widely used in agricultural production. In addition to the productized nematicides, a number of potential nematode biocontrol agents have also been developed in studies using culture media or secondary metabolites of *Streptomyces* [33]. For example, fervenulin isolated from *Streptomyces* sp. CMU-MH021 and the actinomycin V, X_2_ and D isolated from *S. antibioticus* M7 had nematicidal activity against the *M. incognita* and could inhibit eggs hatching [34,35]. Teleocidin B4 isolated from *Streptomyces* sp. 680560 has nematicidal activity against the pine wood nematode, *Bursaphelenchus xylophilus*, and could inhibit eggs hatching [36]. These studies have provided effective alternatives to chemical nematicides for controlling PPN diseases. In this study, we used the model organism *C. elegans* as a drug screening tool to identify the nematicidal compound spectinabilin from *Streptomyces* sp. DT10.

*Streptomyces* have been reported to produce secondary metabolites with a variety of biological activities, and some are powerful biocontrol strains against parasitic nematodes and fungi [18,37,38]. *S. spectabilis* can produce several natural products, among which spectinabilin is a potent nematicide for pine wood nematodes [15,16]. In our study, strain DT10 with nematicidal activity was identified as *Streptomyces* sp. During the process of nematicidal compound purification, both Fr.4 and Fr.7 were fractionated using silica gel column chromatography [39,40], which could kill the *C. elegans* worms. Three components with the similar retention time, absorption wavelength and chromogenic reaction were further isolated from Fr.7 (Figure S1). After the activity tracking experiments of these three components, we found that components **2** and **3** had stronger activity than component **1** (Table S2), but it was difficult to obtain enough monomers compound due to their poor stability. Therefore, only compound **1** was identified as spectinabilin [25]. These results suggested that there were a variety of nematicidal active compounds or derivatives with similar chemical structures in *Streptomyces* sp. DT10 secondary metabolites.

Spectinabilin was reported to have different biological activities, such as antiviral [15] and antimalarial [41]. In our study, spectinabilin showed rapid and effective nematicidal activity against *C. elegans* L1 larvae. Furthermore, it exhibited significant efficacy against the PPN *M. incognita* J2s*,* similar to the reports of other nematicides commonly used in agricultural production, such as avermectin, fluopyram and spirotetramat, which have well-defined mechanisms of action [42,43,44]. Although spectinabilin has been reported to have an effect on pine wood nematodes *Bursaphelenchus xylophilus* (IC_50_ at 24 h = 0.84 μg/mL) [18], this study is the first description of the nematicidal activity of spectinabilin on free-living nematode *C. elegans* and PPN *M. incognita*. In addition to the nematicidal activity of spectinabilin, we tested its effects on the motility of *C. elegans* L4 worms for the first time in this study. The WMicrotracker small animal vitality analyzer is useful for studying the mode of action of various known antinematode compounds (such as levamisole and ivermectin) against model organisms *C. elegans* and parasitic nematodes [45]. Our results showed that spectinabilin could effectively reduce the motility of L4 worms. This activity is partly similar to those of organophosphate and carbamate nematicides, which cause muscle paralysis by acting on the acetyl cholinesterase (AChE) and then affecting the normal movement of nematodes [46]. However, these two classes of nematicides primarily paralyze rather than kill the nematodes. In contrast, spectinabilin not only kills, but also paralyzes *C. elegans* worms, which is different from that of the above two classes of nematicides.

Although it was reported that spectinabilin can cause vacuolar death of *B. xylophilus* [18], there is still no report about the molecular mechanism and drug target genes of spectinabilin. The mechanism of action of spectinabilin was further explored in this study. Based on nematicidal activity tests of spectinabilin against mutants of known drug targets, we found that it can kill all mutants of known nematicidal drug target genes tested in this study, as well as the wild type *C*. *elegans*. This result proved that the drug target of spectinabilin is different from those of commonly used nematicides such as levamisole, ivermectin, piperazine, lannate, abamectin and phosphine thiazole. The compound is highly likely to act on a new class of drug target. The mechanism of action of spectinabilin on the motility of nematodes also remains unclear and requires further study. The identification of drug targets of spectinabilin is underway at the molecular level, using forward genetic screening [47] and gene editing methods such as CRISPER-Cas9 [48].

## 4. Materials and Methods

### 4.1. Bacterial Material

The strains to be screened were isolated from the lichen moss sample collected on Dongfeng Road (106.4443° east longitude, 24.8075° north latitude) of Dashiwei, Leye County, Guangxi Zhuang Autonomous Region in January, 2022. The concentration gradient dilution method was used for strain isolation [49]. A 5 g sample was weighed and placed in a conical flask filled with 45 mL sterilized water, and the soil suspension was prepared by shaking at 28 °C and 150 r/min for 30 min. It was then gradually diluted into different concentration gradients of 10^−2^, 10^−3^ and 10^−4^ with sterile water. The suspensions with concentrations of 10^−3^ and 10^−4^ were subsequently coated on TWYA medium (tap water yeast extract agar: 0.25 g yeast, 0.5 g K_2_HPO_4_·7H_2_O, 18 g agar in 1 L water) and incubated at 25 °C for 7 days in a dark box. Single colonies were selected and transferred to ISP2 medium (4 g yeast powder, 4 g glucose, 10 g malt extract, 20 g agar in 1 L water) plates for isolation and purification.

### 4.2. Nematode Strain and Maintenance

*C. elegans* strains used in this study included N2-Bristol (wild type), DA1316 (resistant to ivermectin) [50], VC2937 (resistant to levamisole) [51], CB407 (resistant to piperazine and showed uncoordinated and constrictive behavior) [52], CB6147 (hypersensitive to many drugs) [53], CB933 (resistant to lannate) [54]. The above strains and *Escherichia coli* (OP50) were obtained from the *Caenorhabditis* Genetics Center (CGC) in Minneapolis, MN, USA. The strains were cultured, maintained and assayed at 20 °C (unless otherwise stated) on nematode growth medium (NGM) seeded with *E. coli* (OP50) bacteria according to established methods [55]. All worm strains used in the assay were cultured in the presence of sufficient food to avoid starvation for at least three generations before use. Synchronized populations of worms for all experiments were obtained by filtering a mixed population of properly maintained worms through an 11 μm pore-sized membrane filter (Merck Millipore Ltd., Burlington, MA, USA) using M9 buffer to obtain the L1 larvae, which were subsequently cultured to grow to the L4 stage before use in the assays.

### 4.3. Screening of Strains with Nematicidal Activity

The 161 strains were cultured for 14 days on ISP*2* agar plates at room temperature. At the end of fermentation, the agar cultures were diced and extracted with ethyl acetate (EtOAc)/methanol (MeOH)/acetic acid (HAc) solution (80:15:5, *v*/*v*/*v*) at room temperature [56]. The organic solvents were evaporated, and the extract was dissolved using DMSO to a concentration of 10 μg/μL. *C. elegans* L1 worms were collected and washed with M9 buffer to obtain a suspension, which was then diluted with M9 buffer to a concentration of approximately 50 worms per 20 µL. A total volume of 100 μL of the test system containing 0.1 μg/μL of extract, approximately 50 worms and M9 buffer was added to a 96-well plate. A 1% (*v*/*v*) DMSO was used as the control. The 96-well plate was put in an incubator at 20 °C for 24 h and observed under an inverted microscope to count the surviving and dead worms. The worms that moved freely or responded by moving the head or tail when the 96-well plate was shaken were considered viable. The mortality was counted as the number of dead worms divided by the total number of worms.

### 4.4. Identification of Strain DT10

Strain DT10 was inoculated on ISP2 medium and cultured at 25 °C for 14 days to observe its growth. The mycelium morphology was observed under the microscope using 40X objective. Genomic DNA was extracted using a TIANGEN kit (Beijing TIANGEN Biochemical Technology Co., Ltd., Beijing, China) and the 16S rRNA sequence was amplified using universal primers 27F(5′-AGAGTTTGATCCTGGCTCAG-3′) and 1495R(5′-CTACGGCTACCTTGTTACGA-3′). The PCR system was 25 μL, containing 12.5 μL 2 × *Taq* PCR Mix, 1 μL 27F and 1 μL 1495R, 9.5 μL ddH_2_O and 1 μL DNA template. The amplification program consisted of predenaturation at 94 °C for 3 min, followed by 35 cycles of denaturation at 94 °C for 30 s, annealing at 60 °C for 30 s and extension at 72 °C for 2.5 min, then the final extension at 72 °C for 8 min. The PCR products were detected using agarose gel electrophoresis, purified and sent to Sangon Bioengineering (Shanghai, China) for sequencing. The sequencing results were compared with the gene sequence in NCBI BLAST, and the sequences with high similarity were downloaded. The phylogenetic tree was constructed using the software MEGA-X [57,58].

### 4.5. Cultivation, Extraction and Isolation

Strain DT10 (2 L) was cultured on ISP2 medium at 25 °C for 14 days. Then, the solid fermentation products of strain DT10 were cut into small pieces and exhaustively extracted using an ethyl acetate(EtOAc)/methanol(MeOH)/acetic acid (HAc) solution (80:15:5, *v*/*v*/*v*) [56]. The extraction was performed three times to generate a crude extract. The extracts were dissolved in water and extracted three times with EtOAc. The residue of EtOAc extract (4.75 g) was subjected to a silica gel G column (Qingdao Marine Chemical Factory) with a length and diameter of 22 × 3 cm, and a silica gel mass of 28 g. A petroleum ether-EtOAc (90:10, 80:20, 70:30, 60:40) and CHCl_3_-MeOH (20:1, 0:100) gradient solvent system was used to produce 9 fractions (Fr.1-Fr.9). Upon completion of activity tracking experiments, it was demonstrated that Fr.4 and Fr.7 possessed a nematicidal effect with a working concentration of 0.2 μg/μL and 0.05 μg/μL, leading to the complete mortality of all nematodes within the experimental cohort within 24 h. Consequently, Fr.7 (594.3 mg) was subjected to semipreparative HPLC purification utilizing MeOH/H_2_O (80:20, 25 min) as the eluent, ultimately resulting in the isolation of compound 1.

### 4.6. Structure Identification of Compound ***1***

The Nuclear Magnetic Resonance (NMR) spectra were recorded using an Agilent NMR system 800 MHz NMR spectrometer (Agilent Technologies Inc., Colorado Springs, CO, USA) to determine the structure of compound **1**. Electrospray ionization mass spectrometry (ESI-MS) and high-resolution electrospray ionization mass spectrometry (HR-ESI-MS) were performed using a Waters Xevo G2-S QTOF MS spectrometer (Waters, Milford, MA, USA) to confirm the molecular weight and structure information of compound **1** isolated from strain DT10.

### 4.7. Effect of Spectinabilin on Survival of C. elegans

The effect of the spectinabilin on the survival of wild type *C. elegans* was tested by incubating the worms in different concentrations of the compound. Worms were washed off the NGM plate with 1 mL of M9 buffer, which contained a mixture of developmental stages. The *C. elegans* L1 worms were collected, washed with M9 buffer to obtain the suspension, which was then diluted with M9 buffer to a concentration of approximately 50 worms per 20 µL. A total volume of 100 μL of the test system containing the set concentration of compound, approximately 50 nematodes and M9 was added to the 96-well plate. A 1% (*v*/*v*) DMSO was used as the control, with three replicates in each group. Nematodes were incubated in an incubator at 20 °C and observed under an inverted microscope to count the surviving and dead nematodes. Nematodes that moved freely or moved the head or tail of their body in response to a shaken 96-well plate were considered viable. Nematode mortality was counted as the number of dead nematodes divided by the total number of nematodes.

### 4.8. Effect of Spectinabilin on Survival of M. incognita J2s

The effect of the spectinabilin on the survival of *M. incognita* J2s was tested by incubating them in different concentrations of the compound. *M. incognita* J2s were collected, washed to obtain the suspension and diluted with sterilized water to a concentration of approximately 50 worms per 20 µL. A total volume of 100 μL of the test system containing 100 μg/mL, 50 μg/mL, 25 μg/mL, 12.5 μg/mL or 6.25 μg/mL of compound, together with 30–50 nematodes and sterilized water, was added to the 96-well plate. A 1% (*v*/*v*) DMSO was used as the control, with 3 replicates in each group. The nematodes were incubated in an incubator at 20 °C and observed under an inverted microscope to count the surviving and dead nematodes. Nematodes that moved freely or moved the head or tail of their body in response to a shaken 96-well plate were considered viable. Nematode mortality was counted as the number of dead nematodes divided by the total number of nematodes.

### 4.9. Detection of Locomotor Ability of C. elegans L4 Worms

Synchronized *C*. *elegans* L4 worms were collected and washed to obtain the suspension and then diluted with M9 buffer to a concentration of approximately 50 worms per 20 µL. A total volume of 400 μL of the test system containing 40 μg/mL of compound, about 200 nematodes and M9 buffer was added to the 24-well plate. A 1% (*v*/*v*) DMSO was used as the control, with 4 replicates in the experiment. Worm activity was captured using a WMicroTracker ARENA unit for four hours, and the disturbance of an infrared beam in individual wells was recorded as a worm activity count over a period of 30 min [59]. Relative activity of motility was counted as the number of nematode movements in 30 min of testing divided by the number of nematode movements in the first 30 min.

### 4.10. Analysis of Potential Drug Targets for Spectinabilin

The drug target was investigated by incubating the L1 worms of N2 and mutants of drug target genes in 4 μg/mL spectinabilin. Five mutants of known nematicidal drug target genes (DA1316, VC2937, CB407, CB6147, CB933) were used to test the survival with spectinabilin to identify the potential drug targets of the compound. Worms were washed off the NGM plate with 1 mL of M9 buffer, which contained a mixture of developmental stages. The *C. elegans* L1 worms were collected, washed with M9 buffer to obtain the suspension, which was then diluted with M9 buffer to a concentration of approximately 50 worms per 20 µL. A total volume of 100 μL of the test system containing 4 μg/mL spectinabilin, approximately 50 nematodes and M9 was added to the 96-well plate. The wild type *C. elegans* N2 was used as the control, with three replicates in each group. Nematodes were incubated in an incubator at 20 °C and observed under an inverted microscope to count the surviving and dead nematodes. If the mutant was resistant, it indicated that spectinabilin acted on the known drug target. However, if the mutant died and the mortality was similar to N2, it showed that the drug target of spectinabilin was different from the known target gene in the test.

### 4.11. Statistical Analysis

The raw data obtained from the experiments were analyzed using Graphpad Prism 8.0.2. The results of each experiment were presented as mean ± standard error of mean (SEM) for 3 independent trials. The statistical significance of the differences between the groups in assays was determined using the log-rank test of the Kaplan–Meier survival analysis. The threshold for statistical significance was set at *p* ≤ 0.05.

## 5. Conclusions

Spectinabilin, a compound with nematicidal activity against *C. elegans* and southern root-knot nematode *M. incognita*, was purified and characterized from *Streptomyces* sp. DT10. Spectinabilin showed a good nematicidal effect on *C. elegans* L1 and L4 worms and significantly reduced the motility of *C. elegans* L4 worms. Further analysis revealed that spectinabilin acts on targets different from the currently commonly used nematode drugs such as abamectin and phosphine thiazole. These findings provide new opportunities for developing more efficient and environmentally friendly control measures for parasitic nematodes while addressing the issue of resistance in these organisms.

## Figures and Tables

**Table 1:** Nematicidal activity of spectinabilin against *C. elegans* L1 at 24 h. Data represent experiments conducted in triplicate; mean ± standard error of the mean (SEM).

Concentration (μg/mL)	Mortality (%)
2.5	8.92 ± 4.45
3	38.56 ± 7.42
4	69.45 ± 7.70
5	95.02 ± 2.78
10	100

**Table 2:** Nematicidal activity of spectinabilin against *M. incognita* J2s at 72 h. Data represent experiments conducted in triplicate; mean ± standard error of the mean (SEM).

Concentration (μg/mL)	Mortality (%)
6.25	0
12.5	12.31 ± 1.72
25	19.20 ± 3.73
50	22.97 ± 2.67
100	40.81 ± 4.44

## Data Availability

The data presented in this study are available in the article.

## Supplementary Materials

The following supporting information can be downloaded at https://www.mdpi.com/article/10.3390/molecules28114365/s1, Figure S1: HPLC spectrum of Fraction 7; Figure S2: HR-ESI-MS spectrum of compound **1**; Figure S3: ^1^H-NMR (800 MHz, MeOD) spectrum of compound **1**; Figure S4: ^13^C-NMR spectrum (200 MHz, MeOD)of compound **1**; Table S1: Screening of nematicidal activity of 161 actinomyces; Table S2: Nematicidal activity of components **1**, **2**, **3** against *C. elegans* L1 after 24 h at 0.1 μg/μL of components; Table S3: Nematicidal activity of spectinabilin against *C. elegans* L1 after 2 h at different concentrations of compound; Table S4: Nematicidal activity of spectinabilin against *C. elegans* L4 after 4 h at different concentrations.

## References

[1] Abd-Elgawad M.M.M. (2021). Optimizing safe approaches to manage plant-parasitic nematodes. Plants.

[2] Tileubayeva Z., Avdeenko A., Avdeenko S., Stroiteleva N., Kondrashev S. (2021). Plant-parasitic nematodes affecting vegetable crops in greenhouses. Saudi J. Biol. Sci..

[3] Abad P., Gouzy J., Aury J.M., Castagnone-Sereno P., Danchin E.G., Deleury E., Perfus-Barbeoch L., Anthouard V., Artiguenave F., Blok V.C. (2008). Genome sequence of the metazoan plant-parasitic nematode *Meloidogyne incognita*. Nat. Biotechnol..

[4] Palomares-Rius J.E., Hasegawa K., Siddique S., Vicente C.S.L. (2021). Editorial: Protecting our crops-approaches for plant parasitic nematode control. Front. Plant Sci..

[5] Martinez-Servat S., Pinyol-Escala L., Daura-Pich O., Almazan M., Hernandez I., Lopez-Garcia B., Fernandez C. (2023). Characterization of *Lysobacter enzymogenes* B25, a potential biological control agent of plant-parasitic nematodes, and its mode of action. AIMS Microbiol..

[6] Caboni P., Ntalli N.G., Aissani N., Cavoski I., Angioni A. (2012). Nematicidal activity of (E, E)-2,4-decadienal and (E)-2-decenal from *Ailanthus altissima* against *Meloidogyne javanica*. J. Agric. Food. Chem..

[7] Geary T.G. (2005). Ivermectin 20 years on: Maturation of a wonder drug. Trends Parasitol..

[8] Lahm G.P., Desaeger J., Smith B.K., Pahutski T.F., Rivera M.A., Meloro T., Kucharczyk R., Lett R.M., Daly A., Smith B.T. (2017). The discovery of fluazaindolizine: A new product for the control of plant parasitic nematodes. Bioorganic Med. Chem. Lett..

[9] Chen J., Song B. (2021). Natural nematicidal active compounds: Recent research progress and outlook. J. Integr. Agric..

[10] Wang L., Yang B., Li C. (2002). A review of biological control of root-knot nematodes. J. Nanjing Fores. U..

[11] János B. (2005). Bioactive Microbial Metabolites. J. Antibiot..

[12] Garabedian S., Van Gundy S.D. (1983). Use of avermectins for the control of *Meloidogyne incognita* on tomatoes. J. Nematol..

[13] Sun Y., Zhou X., Liu J., Bao K., Zhang G., Tu G., Kieser T., Deng Z. (2002). ‘*Streptomyces nanchangensis*’, a producer of the insecticidal polyether antibiotic nanchangmycin and the antiparasitic macrolide meilingmycin, contains multiple polyketide gene clusters. Microbiology.

[14] Engle P. (1987). Plasmid transformation of *Streptomyces tendae* after heat attenuation of restriction. Appl. Environ. Microbiol..

[15] Kakinuma K., Hanson C.A., Rinehart K.L. (1976). Spectinabilin, a new nitro-containing metabolite isolated from *Streptomyces Spectabilis*. Tetrahedron.

[16] Otoguro K., Liu Z., Fukuda K., Li Y., Iwai Y., Tanaka H., Omura S. (1987). Screening for new nematocidal substances of microbial origin by a new method using the pine wood nematode. J. Antibiot..

[17] Traitcheva N., Jenke-Kodama H., He J., Dittmann E., Hertweck C. (2007). Non-colinear polyketide biosynthesis in the aureothin and neoaureothin pathways: An evolutionary perspective. Chembiochem.

[18] Liu M., Hwang B., Jin C., Li W., Park D.J., Seo S.T., Kim C.J. (2019). Screening, isolation and evaluation of a nematicidal compound from actinomycetes against the pine wood nematode*, Bursaphelenchus xylophilus*. Pest Manag. Sci..

[19] Rajasekharan S.K., Lee J. (2020). Hydropic anthelmintics against parasitic nematodes. PLoS Pathog..

[20] Holden-Dye L., Walker R.J. (2014). Anthelmintic drugs and nematicides: Studies in *Caenorhabditis elegans*. WormBook.

[21] Geary T.G., Thompson D.P. (2001). *Caenorhabditis elegans*: How good a model for veterinary parasites?. Vet. Parasitol..

[22] Hahnel S.R., Dilks C.M., Heisler I., Andersen E.C., Kulke D. (2020). *Caenorhabditis elegans* in anthelmintic research—Old model, new perspectives. Int. J. Parasitol. Drugs Drug Resist..

[23] Sepulveda-Crespo D., Reguera R.M., Rojo-Vazquez F., Balana-Fouce R., Martinez-Valladares M. (2020). Drug discovery technologies: *Caenorhabditis elegans* as a model for anthelmintic therapeutics. Med. Res. Rev..

[24] Holden-Dye L., Crisford A., Welz C., von Samson-Himmelstjerna G., Walker R.J., O’connor V. (2012). Worms take to the slo lane: A perspective on the mode of action of emodepside. Invertebr. Neurosci..

[25] Lu C., Zhang Z.Q., Zhang X.C., Kang Q.J. (2020). New spectinabilin and hexadienamide derivatives from *Streptomyces* sp. S012. Rec. Nat. Prod..

[26] Sanadhya P., Bucki P., Liarzi O., Ezra D., Gamliel A., Braun Miyara S. (2018). *Caenorhabditis elegans* susceptibility to *Daldinia* cf. *concentrica* bioactive volatiles is coupled with expression activation of the stress-response transcription factor *daf-16*, a part of distinct nematicidal action. PLoS ONE.

[27] Yan J., Wang X., Tian M., Liu C., Zhang K., Li G. (2017). Chemical constituents from the fungus *Stereum* sp. YMF1.04183. Phytochem. Lett..

[28] Rupcic Z., Chepkirui C., Hernandez-Restrepo M., Crous P.W., Luangsa-Ard J.J., Stadler M. (2018). New nematicidal and antimicrobial secondary metabolites from a new species in the new genus *Pseudobambusicola thailandica*. MycoKeys.

[29] Gupta R., Singh A., Ajayakumar P.V., Pandey R. (2017). Microbial interference mitigates *Meloidogyne incognita* mediated oxidative stress and augments bacoside content in *Bacopa monnieri* L. Microbiol. Res..

[30] Gomez-Escribano J.P., Bibb M.J. (2014). Heterologous expression of natural product biosynthetic gene clusters in *Streptomyces* coelicolor: From genome mining to manipulation of biosynthetic pathways. J. Ind. Microbiol. Biotechnol..

[31] Law J.W., Law L.N., Letchumanan V., Tan L.T., Wong S.H., Chan K.G., Ab Mutalib N.S., Lee L.H. (2020). Anticancer drug discovery from microbial sources: The unique mangrove *Streptomycetes*. Molecules.

[32] Huang X., Li S., Tan Z., Xie B., Wu C., Yang Y. (2008). Progress of study on endophytic actinomycetes in plant. Biotech. Bull..

[33] Kaur T., Manhas R.K. (2014). Antifungal, insecticidal, and plant growth promoting potential of *Streptomyces hydrogenans* DH16. J. Basic. Microbiol..

[34] Ruanpanun P., Laatsch H., Tangchitsomkid N., Lumyong S. (2011). Nematicidal activity of fervenulin isolated from a nematicidal actinomycete, *Streptomyces* sp. CMU-MH021, on *Meloidogyne incognita*. World J. Microbiol. Biotechnol..

[35] Sharma M., Jasrotia S., Ohri P., Manhas R.K. (2019). Nematicidal potential of *Streptomyces antibioticus* strain M7 against *Meloidogyne incognita*. AMB Express.

[36] Kang M.K., Kim M.H., Li M.J., Ji C.Z., Park S.H., Lee J.M., Kim J., Park D.J., Park H.R., Kim Y.H. (2021). Nematicidal activity of teleocidin B4 isolated from *Streptomyces* sp. against pine wood nematode, *Bursaphelenchus xylophilus*. Pest Manag. Sci..

[37] Zhang Z., Wu X., Song R., Zhang J., Wang H., Zhu J., Lu C., Shen Y. (2017). Ansavaricins F–I, new DNA topoisomerase inhibitors produced by *Streptomyces* sp. S012. RSC Advances.

[38] Nair M.G., Chandra A., Thorogod D.L., Davis R.M.G. (1995). Nematocidal and mosquitocidal aromatic nitro-compounds produced by *Streptomyces* spp.. Pestic. Sci..

[39] Zhan B., Zeng Q., Wu X., Zhu J., Bao S., Huang H. (2010). Identification of nematicidal actinomycetes DA09202 and active compounds. Microbiolo. China.

[40] Zheng Y., Pang H., Wang J., Chen D., Shi G., Huang J. (2014). Novel diketopiperazine and ten-membered macrolides from the entomogenous fungus *Paecilomyces tenuipes*. Chem. J. Chin. U..

[41] Isaka M., Jaturapat A., Kramyu J., Tanticharoen M., Thebtaranonth Y. (2002). Potent in vitro antimalarial activity of metacycloprodigiosin isolated from *Streptomyces spectabilis* BCC 4785. Antimicrob. Agents Chemother..

[42] Cully D.F., Vassilatis D.K., Liu K.K., Paress P.S., Van der Ploeg L.H.T., Schaeffer J.M., Arena J.P. (1994). Cloning of an avermectin-sensitive glutamate-gated chloride channel from *Caenorhabditis elegans*. Nature.

[43] Liu Y., Zhang W., Wang Y., Liu H., Zhang S., Ji X., Qiao K. (2022). Oxidative stress, intestinal damage, and cell apoptosis: Toxicity induced by fluopyram in *Caenorhabditis elegans*. Chemosphere.

[44] Vang L.E., Opperman C.H., Schwarz M.R., Davis E.L. (2016). Spirotetramat causes an arrest of nematode juvenile development. Nematology.

[45] Gunderson E., Bulman C., Luo M., Sakanari J. (2020). In Vitro Screening Methods for Parasites: The WMicroTracker & the WormAssay. MicroPubl. Biol..

[46] Opperman C.H., Chang S. (1992). Nematode acetylcholinesterases: Molecular forms and their potential role in nematode behavior. Parasitol. Today.

[47] Jorgensen E.M., Mango S.E. (2002). The art and design of genetic screens: *Caenorhabditis elegans*. Nat. Rev. Genet..

[48] Waaijers S., Boxem M. (2014). Engineering the *Caenorhabditis elegans* genome with CRISPR/Cas9. Methods.

[49] Chen Y., Huang H., Liu M., Bao S. (2014). Isolation and identification of an actinomycetes strain against rootknot nematode from mangrove soil. Biotech. Bull..

[50] Nguyen V.T., Park A.R., Duraisamy K., Vo D.D., Kim J.C. (2022). Elucidation of the nematicidal mode of action of grammicin on *Caenorhabditis elegans*. Pestic. Biochem. Physiol..

[51] Sloan M.A., Reaves B.J., Maclean M.J., Storey B.E., Wolstenholme A.J. (2015). Expression of nicotinic acetylcholine receptor subunits from parasitic nematodes in *Caenorhabditis elegans*. Mol. Biochem. Parasitol..

[52] Câmara D.F., Machado M.L., Arantes L.P., Silva T.C., Silveira T.L., Leal J.G., Dornelles L., Stefanello S.T., Soares F.A. (2019). MPMT-OX up-regulates GABAergic transmission and protects against seizure-like behavior in Caenorhabditis elegans. Neurotoxicology.

[53] WormBase. https://wormbase.org/species/c_elegans/strain/WBStrain00004682#035--10.

[54] Brenner S. (1974). The genetics of *Caenorhabditis elegans*. Genetics.

[55] Sulston J., Hodgkin J. (1988). The Nematode Caenorhabditis Elegans.

[56] Singh P., Xie J., Qi Y., Qin Q., Jin C., Wang B., Fang W. (2021). A thermotolerant marine *Bacillus amyloliquefaciens* S185 producing iturin A5 for antifungal activity against *Fusarium oxysporum* f. sp. *cubense*. Mar. Drugs.

[57] Zheng W., Li D., Zhao J., Liu C., Zhao Y., Xiang W., Wang X. (2017). *Promicromonospora soli* sp. nov., a novel actinomycete isolated from soil. Int. J. Syst. Evol. Microbiol..

[58] Tamura K., Stecher G., Kumar S. (2021). MEGA11: Molecular Evolutionary Genetics Analysis Version 11. Mol. Biol. Evol..

[59] Shanley H.T., Taki A.C., Byrne J.J., Jabbar A., Wells T.N.C., Samby K., Boag P.R., Nguyen N., Sleebs B.E., Gasser R.B. (2022). A high-throughput phenotypic screen of the ‘Pandemic Response Box’ identifies a quinoline derivative with significant anthelmintic activity. Pharmaceuticals.

