# Supplementary material for: Isolation, Identification and Molecular Mechanism Analysis of the Nematicidal Compound Spectinabilin from Newly Isolated Streptomyces sp. DT10
Source: Molecules. 2023 May 26;28(11):4365. doi: 10.3390/molecules28114365 (PMC10254515; doi:10.3390/molecules28114365)
Supplement: Supplementary file 1 [file molecules-28-04365-s001.zip › molecules-2415383-supplementary.pdf]

Supplementary Figures

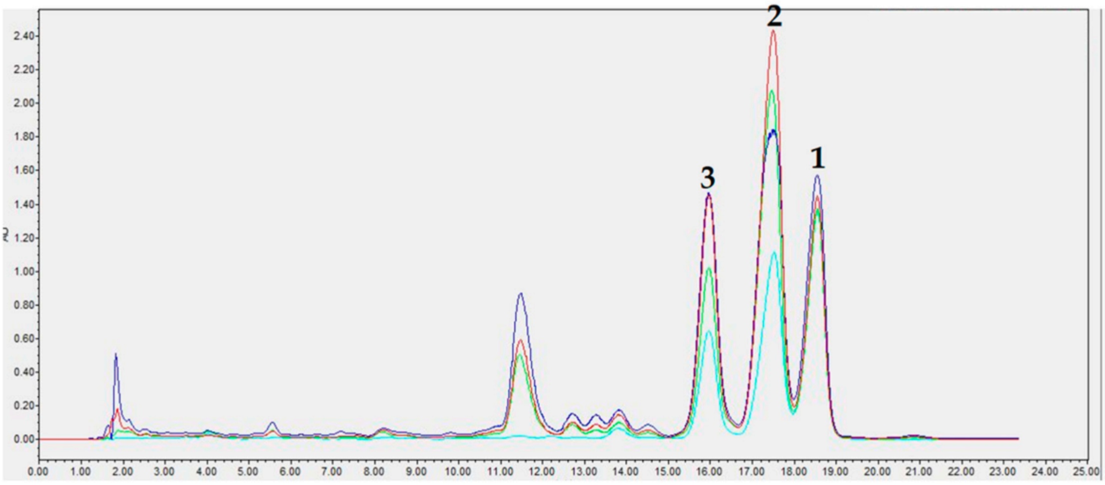

Figure S1: HPLC spectrum of Fraction 7. Fr.7 was subjected to semipreparative HPLC purification utilizing MeOH/H2O (80:20, 25 min) as the eluent, ultimately resulting in the isolation of compound **1**.

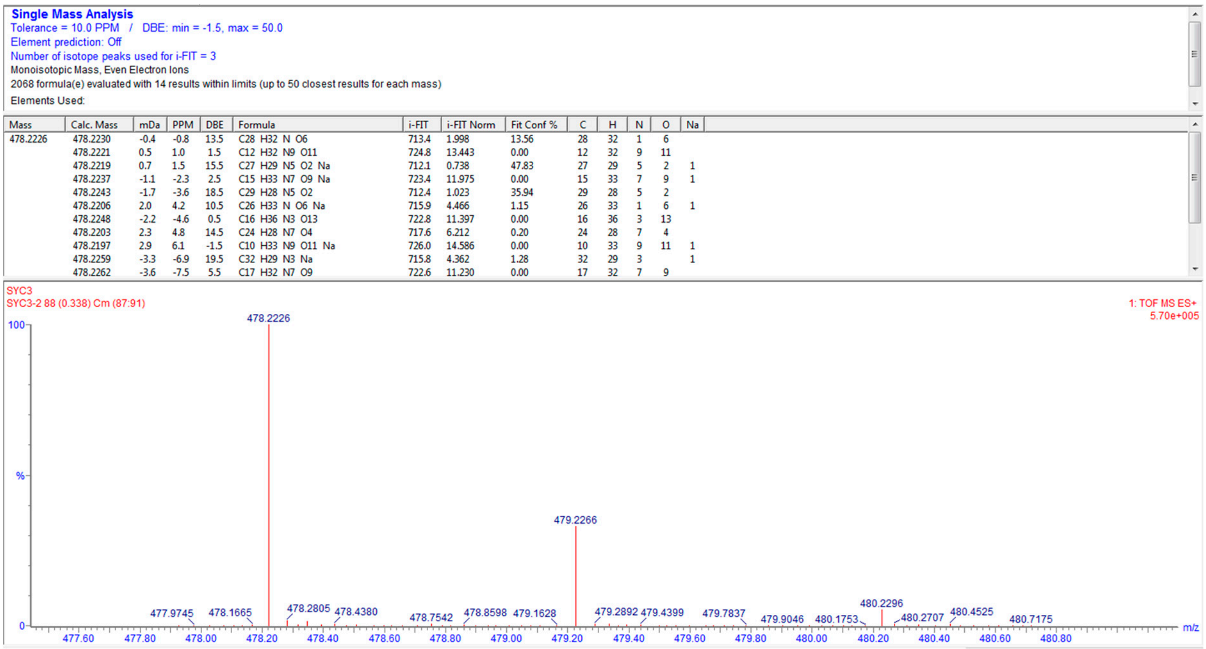

Single Mass Analysis
Tolerance = 10.0 PPM / DBE: min = -1.5, max = 50.0
Element prediction: Off
Number of isotope peaks used for i-FIT = 3
Monoisotopic Mass, Even Electron Ions
2068 formula(e) evaluated with 14 results within limits (up to 50 closest results for each mass)
Elements Used:

Mass	Calc. Mass	mDa	PPM	DBE	Formula	i-FIT	i-FIT Norm	Fit Conf %	C	H	N	O	Na
478.2226	478.2230	-0.4	-0.8	13.5	C28 H32 N O6	713.4	1.998	13.56	28	32	1	6	
	478.2221	0.5	1.0	1.5	C12 H32 N9 O11	724.8	13.443	0.00	12	32	9	11	
	478.2219	0.7	1.5	15.5	C27 H29 N5 O2 Na	712.1	0.738	47.83	27	29	5	2	1
	478.2237	-1.1	-2.3	2.5	C15 H33 N7 O9 Na	723.4	11.975	0.00	15	33	7	9	1
	478.2243	-1.7	-3.6	18.5	C29 H28 N5 O2	712.4	1.023	35.94	29	28	5	2	
	478.2206	2.0	4.2	10.5	C26 H33 N O6 Na	715.9	4.466	1.15	26	33	1	6	1
	478.2248	-2.2	-4.6	0.5	C16 H36 N3 O13	722.8	11.397	0.00	16	36	3	13	
	478.2203	2.3	4.8	14.5	C24 H28 N7 O4	717.6	6.212	0.20	24	28	7	4	
	478.2197	2.9	6.1	-1.5	C10 H33 N9 O11 Na	726.0	14.586	0.00	10	33	9	11	1
	478.2259	-3.3	-6.9	19.5	C32 H29 N3 Na	715.8	4.362	1.28	32	29	3		1
	478.2262	-3.6	-7.5	5.5	C17 H32 N7 O9	722.6	11.230	0.00	17	32	7	9	

Figure S2: HR-ESI-MS spectrum of compound **1**.

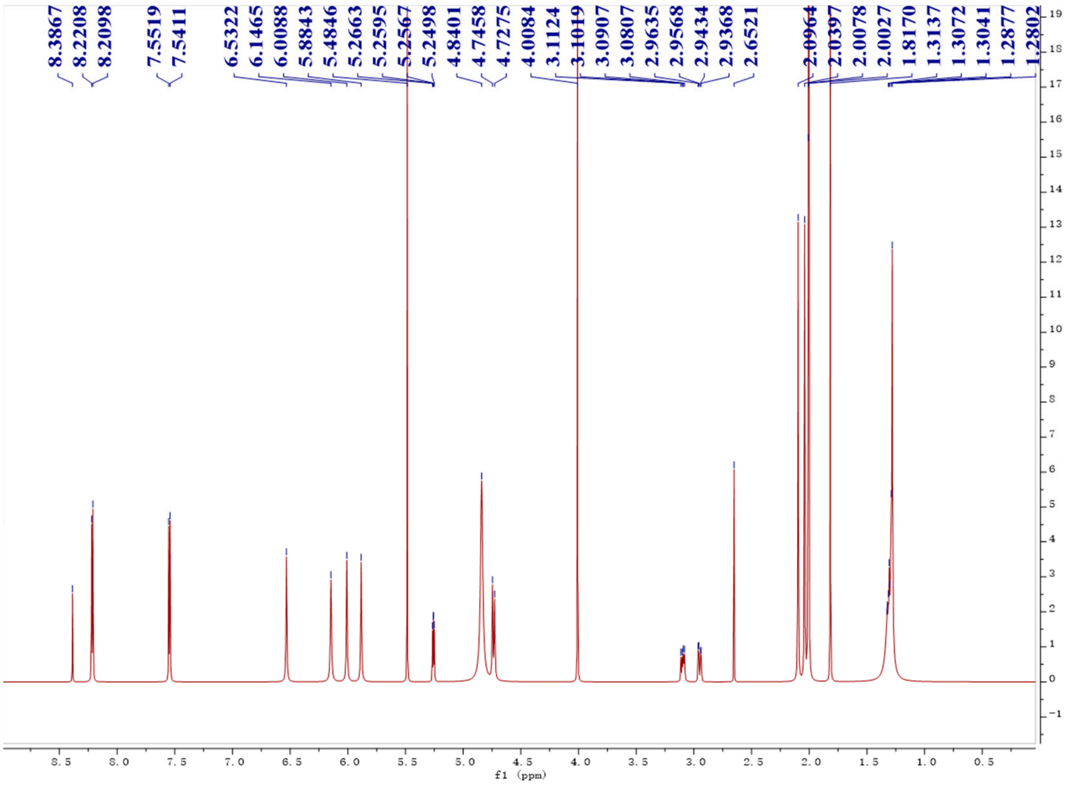

Figure S3: ^{1}H-NMR(800 MHz, MeOD) spectrum of compound **1**.

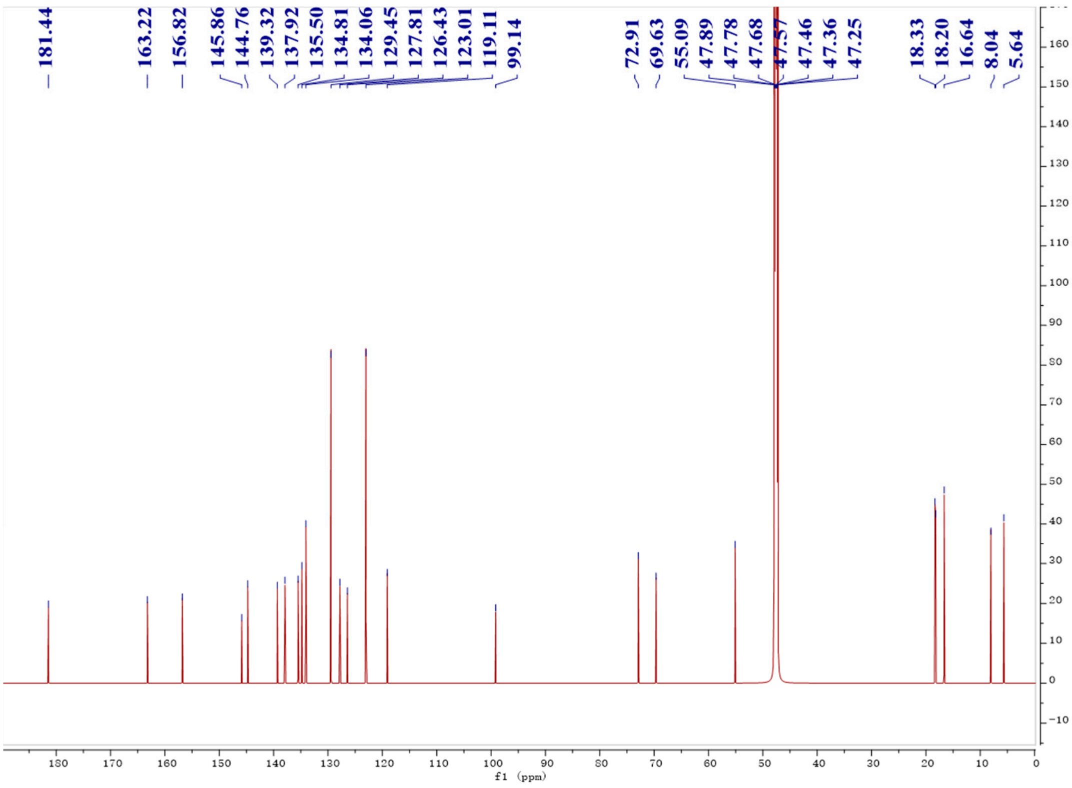

Figure S4: ^{13}C-NMR spectrum (200 MHz, MeOD)of compound **1**.

Supplementary Tables

Table S1. Screening of nematicidal activity of 161 actinomyces. The crude extractions from 161 actinomyces were tested against *C.elegans* for nematicidal activity at 100 μg/mL after 24 h. Actinomyces with nematicidal activity (mortality＞ 80%) were marked with ″**+**″, while others were marked with "**-**".

Table S1a

Strain Number	Nematoidal activity
DG1	-
DG4	-
DG5	-
DG6	-
DI1	-
DI2	-
DI3	-
DI4	-
DI5	-
DI7	-
DI8	-
DI10	-
DI17	-
DT3	-
DT5	-
DT6	-
DT8	-
DT9	-
DT10	+
DT11	-
DT12	-
DT13	-
DT15	-
DH2	-
DH3	-
DH5	-
DH6	-
DH7	+
YG3	-
YG5	-

Table S1b

Strain Number	Nematoidal activity
YG11	-
YG17	-
YG19	-
YG20	-
YG22	-
YG23	-
YG24	-
YG25	-
YG27	-
YG28	-
YG30	-
YG34	-
YG36	-
YG37	-
YG38	-
YG39	-
YG40	-
YG41	-
YG42	-
YG43	-
YG44	-
YG45	-
YG46	-
YG47	-
YG48	-
YG49	-
YG50	-
YI3	-
YI5	-
YI6	-
YI7	-
YI9	-
YI11H	-

Table S1c

Strain Number	Nematoidal activity
YI12	-
YI13	-
YI13H	-
YI14	-
YI16	-
YI17	-
YI20	-
YI21	-
YI22	-
YI23	-
YI24-A	-
YI25	-
YI26	-
YI27	-
YI28	-
YI30	-
YI31	-
YI32	-
YI33	-
YI34	-
YI35	-
YI36	-
YI37	-
YI38	-
YI41-white	-
YI41-brown	-
YI44	-
YI45	-
YI46	-
YI48	-
YI49	-
YI50	-
YI55	-

Table S1d

Strain Number	Nematoidal activity
YI56	-
YI62	-
YI63	-
YI65	-
YI71	-
YI72	-
YI73	-
YI74	-
YI75	-
YI76	-
YI77	-
YI78	-
YI80	-
YI81	-
YI82	-
YI83	-
YI84	-
YI90	-
YI92	-
YI94	-
YI97	-
YI98	-
YI101	-
YI102	-
YI109	-
YH1	-
YH3	-
YH4	-
YH8	-
YH9	-
YH11	-
YH12	-
YH14	-

Table S1e

Strain Number	Nematoidal activity
YT1A	-
YT1B	-
YT3	-
YT4	-
YT5	-
YT6	-
YT8	-
YT9A	-
YT9B	-
YT10	-
YT11	+
YT12	-
YT16	-
YT18	-
YT22	-
YT26	-
YT27	-
YT28	-
YT29	-
YT30	-
YT33	-
YT34	-
YT36	-
YT37	-
YT38	-
YT39	-
YT41	-
YT42	-
YT44	-
YT45	-
YT98	-

Table S2. Nematicidal activity of components 1, 2, 3 against *C.elegans* L1 after 24 h at the concentration of 0.1 μg/μL. Data represent one experiment.

Components (No.)	Mortality/%
1	80.00
2	93.10
3	93.94

Table S3. Nematicidal activity of spectinabilin against *C.elegans* L1 after 2 h at different concentrations of compound. Data represent experiments conducted in triplicate; mean ± standard error of the mean (SEM).

Concentration/(μg/mL)	Mortality/%
3.125	13.90±1.00
6.25	19.16±1.25
12.5	32.74±1.75
25	72.38±4.32
50	85.32±2.58
100	94.06±3.22
200	97.13±2.28
400	100

Table S4. Nematicidal activity of spectinabilin against *C. elegans* L4 after 4 h at different concentrations. Data represent experiments conducted in triplicate; mean±standard error of the mean (SEM).

Concentration/(μg/mL)	Mortality/%
5	0
10	0
20	6.68±0.82
40	12.70±2.19
80	35.04±2.00
